# Ichthyoplankton assemblage structure of springs in the Yangtze Estuary revealed by biological and environmental visions

**DOI:** 10.7717/peerj.1186

**Published:** 2015-08-18

**Authors:** Hui Zhang, Weiwei Xian, Shude Liu

**Affiliations:** Key Laboratory of Marine Ecology and Environment Sciences, Institute of Oceanology, Chinese Academy of Sciences, Qingdao, China

**Keywords:** Ichthyoplankton, Assemblage structure, Environment, Inter-annual variation, Yangtze Estuary

## Abstract

The ichthyoplankton assemblage structure in the Yangtze Estuary was analyzed based on four springs in 1999, 2001, 2004 and 2007 in order to provide detailed characterizations of the ichthyoplankton assemblage in springs, examine the long-term dynamics of spring ichthyoplankton assemblages, and evaluate the influence of environmental factors on the spatial distribution and inter-annual variations of ichthyoplankton assemblages associated with the Yangtze Estuary. Forty-two ichthyoplankton species belonging to 23 families were collected. Engraulidae was the most abundant family, including six species and comprising 67.91% of the total catch. Only four species (*Coilia mystus*, *Engraulis japonicus*, *Trachidermis fasciatus* and *Allanetta bleekeri*) could be considered dominant, accounting for 88.70% of total abundance. The structure of the ichthyoplankton spring assemblage persisted on an annual basis, with the dominant species reappearing consistently even though their abundance fluctuated from year to year. This inter-annual variation probably reflects variable environmental conditions influenced by jellyfish blooms, declining river flow, and overfishing. Canonical correspondence analysis indicated aspatial structure of the ichthyoplankton assemblage in three areas: (1) an inner assemblage dominated by *C. mystus*; (2) a central assemblage dominated by *A. bleekeri* and *T. fasciatus*; and (3) a shelf assemblage featuring *E. japonicus*. The observed ichthyoplankton assemblage structure appears to be strongly influenced by depth, salinity and suspended particulate matter gradients.

## Introduction

An estuary is a partially enclosed coastal body of water. It is either permanently or periodically open to the sea, and there is a measurable variation in salinity due to the mixture of seawater with fresh water derived from land drainage (Day, 1980). Being transition zones between seas and freshwater, estuaries play an important role not only in transport, industry and tourism but also in drainage of waste from domestic, industrial and agriculture activities (Raz-Guzman & Huidobro, 2002). In particular, estuaries support high abundances of organisms owing to their high productivity, providing important nursery areas where ichthyoplankton encounter suitable conditions for enhanced development (Moyle & Cech, 1996).

The important roles of estuaries in the early stages of fish ontogenesis are well documented. For example, abundant food supply facilitates the rapid growth of juveniles (Whitfield, 1999); the level of predation on juveniles in estuaries is presumably reduced owing to a lower incidence of predators compared with their natal marine environment and the protection provided by macrophyte beds or turbid waters often found in these systems (Potter et al., 1990; Whitfield, 1999; Islam, Hibino & Tanaka, 2006). Higher spring/summer water temperature in estuaries compared with the sea will also help growth of the large numbers of juveniles that enter estuaries at this time (Potter et al., 1990; Neira, Potter & Bradley, 1992).

The ichthyoplankton assemblages in estuaries are complex both in species composition and distribution. Studies show that the organization of ichthyoplankton in estuarine systems is influenced by the interactive effects of a multitude of biotic and abiotic processes. Biological factors include the location, timing and manners of spawning, larval life history, larval behavior, rates of predation, and feeding (Leis, 1991; Azeiteiro et al., 2006). Physical factors include salinity (Whitfield, 1999), temperature (Blaxter, 1992), turbidity (Islam, Hibino & Tanaka, 2006), dissolved oxygen (Rakocinski, LyczkowskiShultz & Richardson, 1996), depth (Wantiez, Hamerlin-Vivien & Kulbicki, 1996), river flow (Faria, Morais & Chícharo, 2006), sediment characteristics and hydrographic events such as currents, winds, eddies, upwelling and stratification of the water column (Gray, 1993).

The Yangtze Estuary is generally considered to be an important nursery habitat and a great number of juvenile fishes use it as a feeding ground and refuge from predation (Luo & Shen, 1994). Previous studies carried out in the Yangtze Estuary have presented evidence on ichthyoplankton assemblage composition, species distribution (Jiang, Shen & Wang, 2006), and the relationship–elucidated elementarily without statistical calculation–between assemblage structure of ichthyoplankton and environmental factors (Zhu, Liu & Sha, 2002; Jiang, Shen & Chen, 2006). However, the study periods in the previous works have been only 1 or 2 years at most. Therefore, there is still confusion about how the Yangtze Estuary’s physical and chemical characteristics affect ichthyoplankton assemblages or control inter-annual variations in estuarine ichthyoplankton.

The Yangtze Estuary has been extensively modified and threatened during the last decades, with numerous projects completed such as the Three Gorges Dam, South-to-North Water Diversion, and Yangtze–Taihu Water Diversion. The projects have considerably reduced rivers flowing to the Estuary, brought heavy contamination to estuarine aquaculture and habitation, and deteriorated water quality. This degradation has in turn led to a need for multi-year comprehensive surveys. The present study was based on surveys at four springs in 1999, 2001, 2004 and 2007. Our aims are to provide detailed characterizations of the ichthyoplankton assemblage in springs, examine the long-term dynamics of spring ichthyoplankton assemblages, and evaluate the influence of environmental factors on the spatial distribution and inter-annual variations of ichthyoplankton assemblages associated with the Yangtze Estuary.

## Materials and Methods

### Samples and data collection

**Figure 1:**
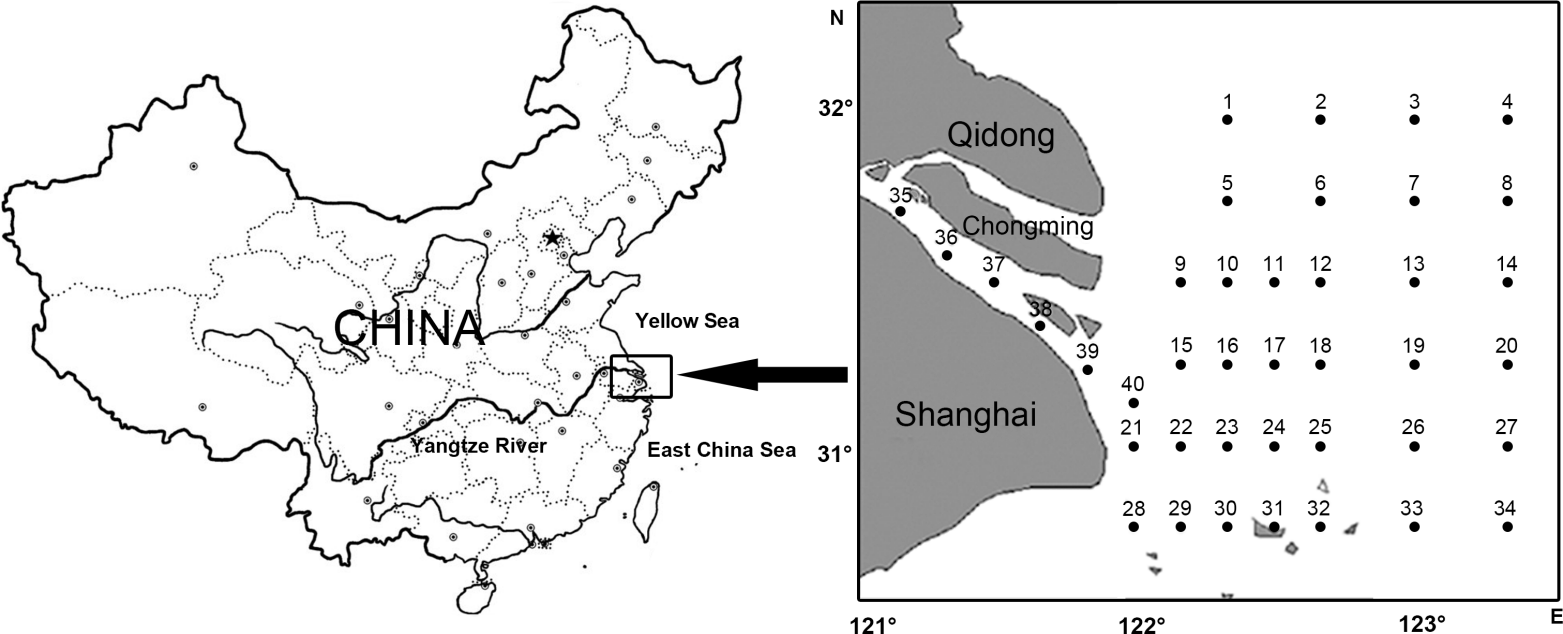
Location of study area and sampling stations of ichthyoplankton in Yangtze Estuary.

Biological and oceanographic data were collected during four fishery evaluation cruises in four springs (05/1999, 05/2001, 05/2004, 05/2007) at 40 stations (Fig. 1).

This study encompasses 160 ichthyoplankton collections from four springs. Ichthyoplankton samples were collected by surface tows of a larva net (0.8 m mouth diameter, 2.8 m long, 0.505 mm mesh at the body, and 0.505 mm mesh at the cod end) equipped with a flow meter. At each station, the net was towed at a depth of about 0.5 m from the surface for 10 min against tidal flow. Towing speed was C.2-3 knots. The samples were preserved in 5% buffered formaldehyde–seawater solution.

A conductivity, temperature, and depth device was used to measure environmental variables including depth (D), salinity (S), temperature (T), transparency (Trans), dissolved oxygen (DO), pH, total phosphorus (TP), total nitrogen (TN), suspended particulate matter (SPM), chemical oxygen demand (COD), chlorophyll *a* (Chla) and primary productivity (PP).

### Data analysis

Ichthyoplankton were identified according to morphological characters. Numerical density for each species was standardized to catch per unit effort (CPUE) as abundance per tow every 10 min. Inter-annual variation of environmental variables and ichthyoplankton abundances were analyzed with a non-parametric Kruskal–Wallis test. A Mann–Whitney test was used for pairwise comparisons.

The dominant species were determined using the Index of Relative Importance (IRI) developed by Zhu, Liu & Sha (2002):

\begin{eqnarray*} \mathrm{IRI}=N\ast 100\% \ast F\ast 100\% \end{eqnarray*}

*N*∗100% and *F*∗100% are the relative abundance and frequency of occurrence, respectively. The IRI of the dominant species should be greater than 100.

To determine the significance of inter-annual trends of the assemblage structure in springs, the non-parametric ANOSIM analysis was conducted, which was based on a Bray–Curtis similarity matrix calculated using log(*x* + 1) transformed data (Clarke & Warwick, 2001). R-statistic values for pair-wise comparisons provided by ANOSIM were used to determine the dissimilarity between groups. Values close to 1 indicate a very different composition, whereas values near to 0 show little difference.

The ichthyoplankton assemblage structure and the relationship of assemblage structure to environmental characteristics were analyzed by canonical correspondence analysis (CCA), which was used to visualize and describe the relationship between fish species and environmental variables (CANOCO Software, Version 4.5). Only species that occurred >1% were included in the analysis. Species abundance data were transformed after lg(*x* + 1) to reduce the dominance effect of some species. Twenty-five species and 12 environmental factors were selected in the CCA analysis in the present study.

## Results

### Ichthyoplankton composition

In total, 8,789 ichthyoplankton individuals were captured. The ichthyoplankton data exemplified a wide taxonomic diversity, as shown by the distribution of 42 identifiable taxa among 23 different families. Dominant taxa included Engraulidae (6 species, 67.91% of the ichthyoplankton), Gobiidae (5 species), Sciaenidae (5 species) and Cynoglossidae (3 species) as shown in Table 1.

**Table 1:** Ichthyoplankton species information.

Family	Species	EG	Percentage	1999		2001		2004		2007	
				F%	A%	F%	A%	F%	A%	F%	A%
Engraulidae	*Engraulis japonicus*	MED	20.15	34.38	**51.10**	32.35	**10.60**	16.67	**11.66**	26.32	**6.70**
	*Coilia mystus*	AN	46.30	31.25	**21.02**	23.53	**58.47**	19.44	**30.06**	21.05	**32.46**
	*Thrissa kammalensis*	MED	1.06			2.94	1.63			2.63	0.15
	*Setipinna taty*	MED	0.01			2.94	0.02				
	*Anchoviella commersoni*	ER	0.33			8.82	0.27	13.89	3.07	5.26	0.58
	*Anchoviella zollingeri*	ER	0.07			2.94	0.11				
Gobiidae	*Chaeturichthys hexanema*	ER	2.86	12.50	**10.98**	5.88	0.28			2.63	0.15
	*Chaeturichthys stigmatias*	ER	2.88			14.71	4.48				
	*Synechogobius hasta*	ER	0.67			11.76	1.05				
	*Gobiidae* sp.	ER	0.28			5.88	0.43			2.63	0.15
Sciaenidae	*Pseudosciaena polyactis*	MED	0.18	6.25	0.28	20.59	0.12			5.26	0.44
	*Larimichthys crocea*	MED	0.01					2.78	0.31		
	*Collichthys lucidus*	MED	0.28			2.94	0.43	2.78	0.31		
	*Johnius belengeri*	MED	0.02			0.00	0.04				
	*Sciaenidae* sp.	MED	0.01			2.94	0.02				
Cynoglossidae	*Cynoglossus abbreviatus*	ER	0.05			2.94	0.07				
	*Cynoglossus joyneri*	ER	0.14			2.94	0.21				
	*Cynoglossus* spp.	ER	0.02			5.88	0.04				
Cyprinidae	*Pseudolaubuca engraulis*	FS	0.01					2.78	0.31		
	*Pseudolaubuca sinensis*	FS	0.03			2.94	0.04	2.78	0.31		
Salangidae	*Hemisalanx prognathus*	AN	0.47	3.13	1.83			5.56	0.61		
	*Salanx ariskensis*	AN	0.02							5.26	0.29
Atherinidae	*Allanetta bleekeri*	MED	8.61	40.63	**7.93**	41.18	3.92	25.00	**30.37**	26.32	**39.01**
Cottidae	*Trachidermis fasciatus*	CA	13.64	12.50	2.77	20.59	**16.69**	11.11	**22.39**	13.16	**18.20**
Stromateidae	*Pampus argenteus*	MED	0.13	3.13	0.23	2.94	0.07			5.26	0.29
Myctophidae	*Benthosema pterotum*	MS	0.85	15.63	3.24	2.94	0.09			2.63	0.15
Apogonidae	*Apogonichthys lineatus*	MS	0.01							2.63	0.15
Mugilidae	*Liza carinatus*	ER	0.06							2.63	0.73
Scombridae	*Pneumatophorus japonicus*	MS	0.08	6.25	0.09	14.71	0.09				
Carangidae	*Trachurus japonicus*	MS	0.01							2.63	0.15
Platycephalidae	*Platycepalus indicus*	MS	0.19	3.13	0.05	8.82	0.28				
Scorpaenidae	*Sebastiscus marmoratus*	MS	0.20			8.82	0.32				
Hemiramphidae	*Hemirhamphus sajori*	MS	0.01							2.63	0.15
Zoarcidae	*Enedrias nebulosus*	MS	0.01					2.78	0.31		
Taeniodididae	*Odontamblyopus rubicundus*	ER	0.01			2.94	0.02				
Fistulariidae	*Fistularia petimba*	MS	0.01			2.94	0.02				
Sparidae	*Sparidae* sp.	MED	0.09	6.25	0.38						
Tetraodontidae	*Taki fugu* sp.	AN	0.08	3.13	0.09	2.94	0.05			5.26	0.29
Anguillidae	*Anguillidae* sp.	CA	0.03			0.00	0.05				
n.id.1		/	0.08			2.94	0.11	2.78	0.31		
n.id.2		/	0.01			2.94	0.02				
n.id.3		/	0.01								

**Notes.**

EG Ecological guilds F% Frequency percentage A% Abundance percentage MS Marine species MED Marine-estuarine-dependents ER Estuarine residents FS Freshwater species CA Catadromous fishes AN Anadromous fishes

The species with high abundance included *Coilia mystus* (46.30%), *Engraulis japonicus* (20.15%), *Trachidermis fasciatus* (13.64%) and *Allanetta bleekeri* (8.61%). Most of the species with low abundance were collected occasionally and not the common species.

### Inter-annual variability of assemblage structure

According to IRI, the dominant species were almost the same across the four springs (Table 2). The ichthyoplankton composition based on presence–absence methods was highly similar among years (ANOSIM, Table 3), and average similarities among years were very high (>75%, Table 3).

**Table 2:** Dominant species determined by the IRI.

1999		2001		2004		2007	
Species	IRI	Species	IRI	Species	IRI	Species	IRI
*E. japonicus*	5,915	*C. mystus*	5,624	*A. bleekeri*	4,131	*A. bleekeri*	4,800
*C. mystus*	2,212	*E. japonicus*	1,783	*C. mystus*	3,180	*C. mystus*	3,200
*A. bleekeri*	1,085	*T. fasciatus*	1,405	*T. fasciatus*	1,354	*T. fasciatus*	1,100
*C. hexanema*	462	*A. bleekeri*	753	*E. japonicus*	1,057	*E. japonicus*	800

**Table 3:** Inter-annual comparison of the assemblage structure according to one-way ANOSIM (*R* value and significance level).

Years	ANOSIM		SIMPER
	*R*	*P*	Average similarity (%)
Global	0.002	0.443	
1999 vs. 2001	0.014	0.235	78.19
1999 vs. 2004	0.007	0.325	75.05
1999 vs. 2007	0.023	0.868	75.12
2001 vs. 2004	0.024	0.193	79.28
2001 vs. 2007	0.006	0.296	79.69
2004 vs. 2007	0.026	0.818	76.05

**Figure 2:**
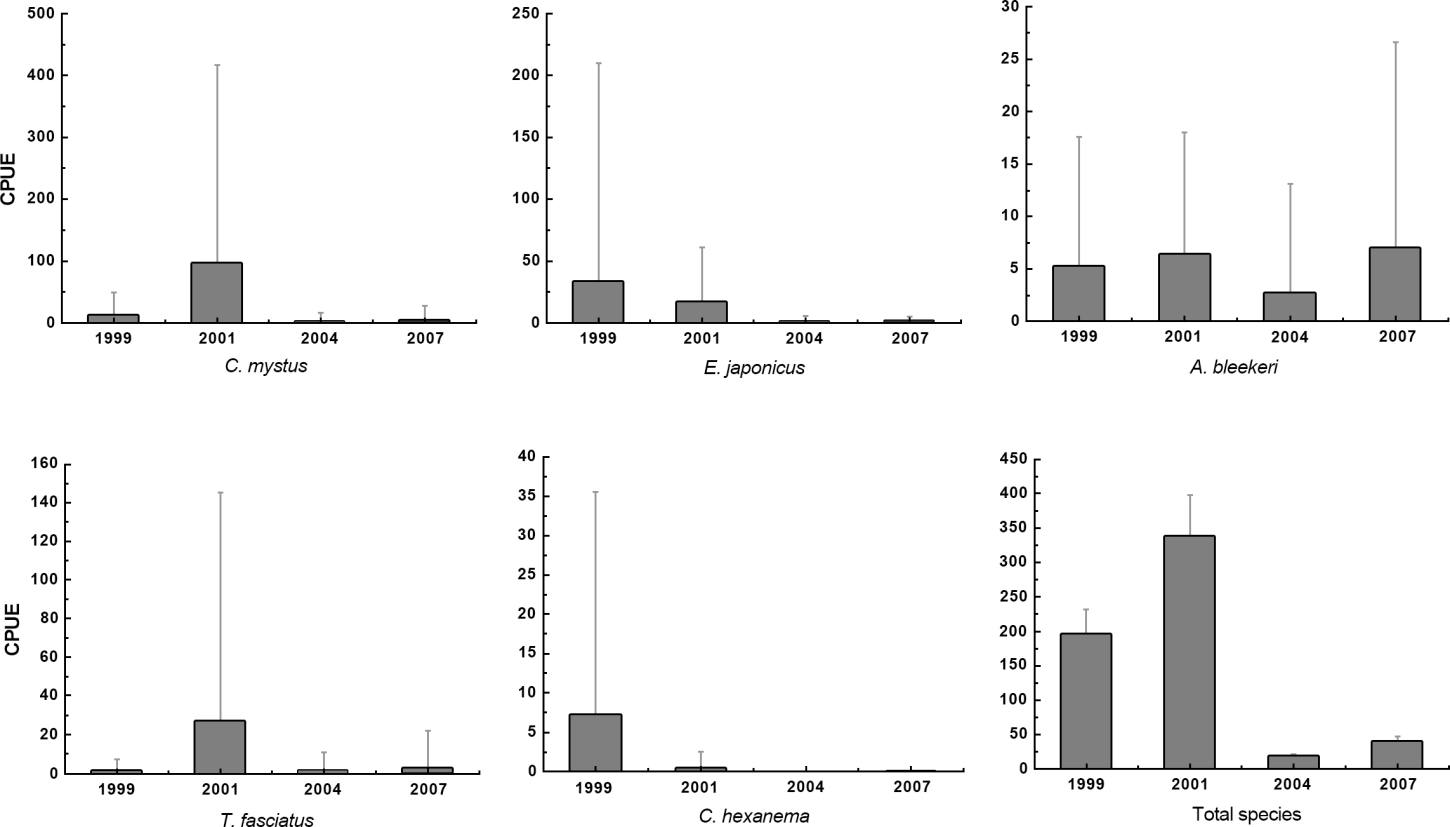
Inter-annual variation CPUE of spring for dominant species in the Yangtze Estuary.

Although no significant inter-annual variation was observed in species composition or assemblage structure over the four springs (ANOSIM, *R* < 0.1, *P* > 0.05; Table 4), the mean CPUE of total species varied significantly on an annual basis (Kruskal–Wallis, *P* < 0.01) (Fig. 2), especially the five dominant species *E. japonicus*, *C. mystus*, *A. bleekeri*, *T. fasciatus* and *Chaeturichthys hexanema* (Fig. 2). However, these inter-annual variations do not follow the same trend for each of the five dominant species. The CPUE for *E. japonicus* and *C. hexanema* declined significantly with time during the spring period (Mann–Whitney, *P* < 0.05). Peak CPUEs of 97.06 ind./tow and 27.71 ind./tow were recorded for *C. mystus* and *T. fasciatus*, respectively, in 2001, while CPUE for *A. bleekeri* peaked in 2007.

**Table 4:** Environmental factors (means and range) in the Yangtze Estuary.

Variables	1999	2001	2004	2007
D (m)	19.52 (5.00–48.00)	20.48 (5.00–50.00)	19.57 (6.00–60.00)	21.45 (5.00–52.00)
S (‰)	15.79 (2.86–27.56)^A^	17.93 (3.00–31.58)^A,B^	20.97 (3.00–30.19)^B^	21.46 (3.00–31.25)^B^
T (°C)	19.19 (14.98–20.86)^A^	18.14 (16.37–21.17)^B^	20.79 (19.15–22.22)^C^	18.77 (17.65–20.53)^D^
DO (mg/L)	9.11 (7.34–13.95)^A^	5.99 (5.14–8.73)^B,C^	6.27 (4.01–12.03)^C^	8.10 (3.90–10.20)^D^
pH	8.29 (7.82–8.85)^A^	8.17 (7.95–8.36)^B^	8.13 (7.94–8.47)^C^	8.39 (7.95–8.71)^C,D^
COD (mg/L)	2.19 (0.77–3.47)	2.06 (0.64–4.32)	1.92 (0.46–4.22)	1.72 (0.73–4.24)
TN (µmol/L)	79.37 (35.80–151.30)^A,C^	60.89 (13.70–77.70)^B^	62.90 (26.30–112.10)^A,B^	90.65 (35.71–176.18)^C^
TP (µmol/L)	1.64 (0.52–6.50)	1.89 (0.30–7.00)	1.47 (0.61–3.50)	1.29 (0.17–4.28)
SPM (mg/L)	24.34 (1.30–95.20)	186.13 (1.30–1,685.00)	67.03 (2.30–298.70)	53.30 (1.20–541.40)
Chla (mg/m^3^)	1.76 (0.21–5.85)	1.83 (0.19–7.41)	2.73 (0.09–17.62)	0.82 (0.21–2.87)
Trans	1.82 (0.20–6.30)	1.57 (0.20–8.50)	1.99 (0.45–6.60)	1.93 (0.30–6.00)
PP (mgC/(m^2^∗d))	536.55 (9.86–2,801.17)^A,B^	389.91 (18.84–1,810.45)^A^	1048.22 (6.70–8,326.82)^A,B^	264.70 (11.84–1,232.01)^B^

**Notes.**

Values with different letters (^A–D^) indicate significant difference among years, and values with the same letter indicate that the difference was not significant.

### Environmental factors

Environmental factors in Yangtze Estuary are shown in Table 4. D of the Yangtze Estuary in the present study ranged from 5 m to 60 m. S increased significantly (*P* < 0.05) and attained the highest value in 2007. T showed significant (*P* < 0.01) inter-annual variations with the lowest value in 2001. DO and pH varied significantly but in a disorderly way among years (*P* < 0.01). COD declined from 1999 to 2007 with no significant variation (*P* > 0.05). Significant inter-annual variations were observed in total nitrogen (TN; *P* < 0.01). SPM was low in 1999 and high in 2001 without significant differences among years. Trans ranged from 1.82 (1999) to 1.93 (2007) without significant differences among years (*P* > 0.05). Chla and primary production (PP) showed the same trends with highest values in 2004 and lowest values in 2007. Significant variations were only found in PP (*P* < 0.05). In all, different environmental factors varied among years but showed different trends.

### Ichthyoplankton assemblages and environmental variables

Eigenvalues indicates the importance of the CCA axes that vary from 0 to 1. In the present study the eigenvalues were 0.490 (CCA 1), 0.33878 (CCA 2), 0.270 (CCA 3) and 0.190 (CCA 4) as shown in Table 5. The eigenvalues of the first two axes were moderately high, whereas the last two were relatively low (<0.3). The sum of all canonical eigenvalues (1.941) only equaled 23.12% of the unconstrained eigenvalues (8.394), showing the restrictive effect of building environmental relationships into the CCA model. For the first four assemblage axes, the cumulative percentage of species variance (CPSV) was 15.3%, and the cumulative percentage of species–environment (CPSE) was 66.4% (Table 5). The first two CCA axes explained 64.71% of the CPSV and 64.16% of the CPSE; therefore, the results for these two axes are plotted (Fig. 2).

**Table 5:** Results of CCA in the present study.

	CCA axes				
	1	2	3	4	Total inertia
Eigenvalues	0.490	0.338	0.270	0.190	8.394
Species-environment correlations	0.811	0.772	0.846	0.623	
Cumulative percentage variance					
of species data	5.8	9.9	13.1	15.3	
of species-environment ralation	25.2	42.6	56.6	66.4	
Sum of all unconstrained eigenvalues					8.394
Sum of all canonical eigenvalues					1.941

**Figure 3:**
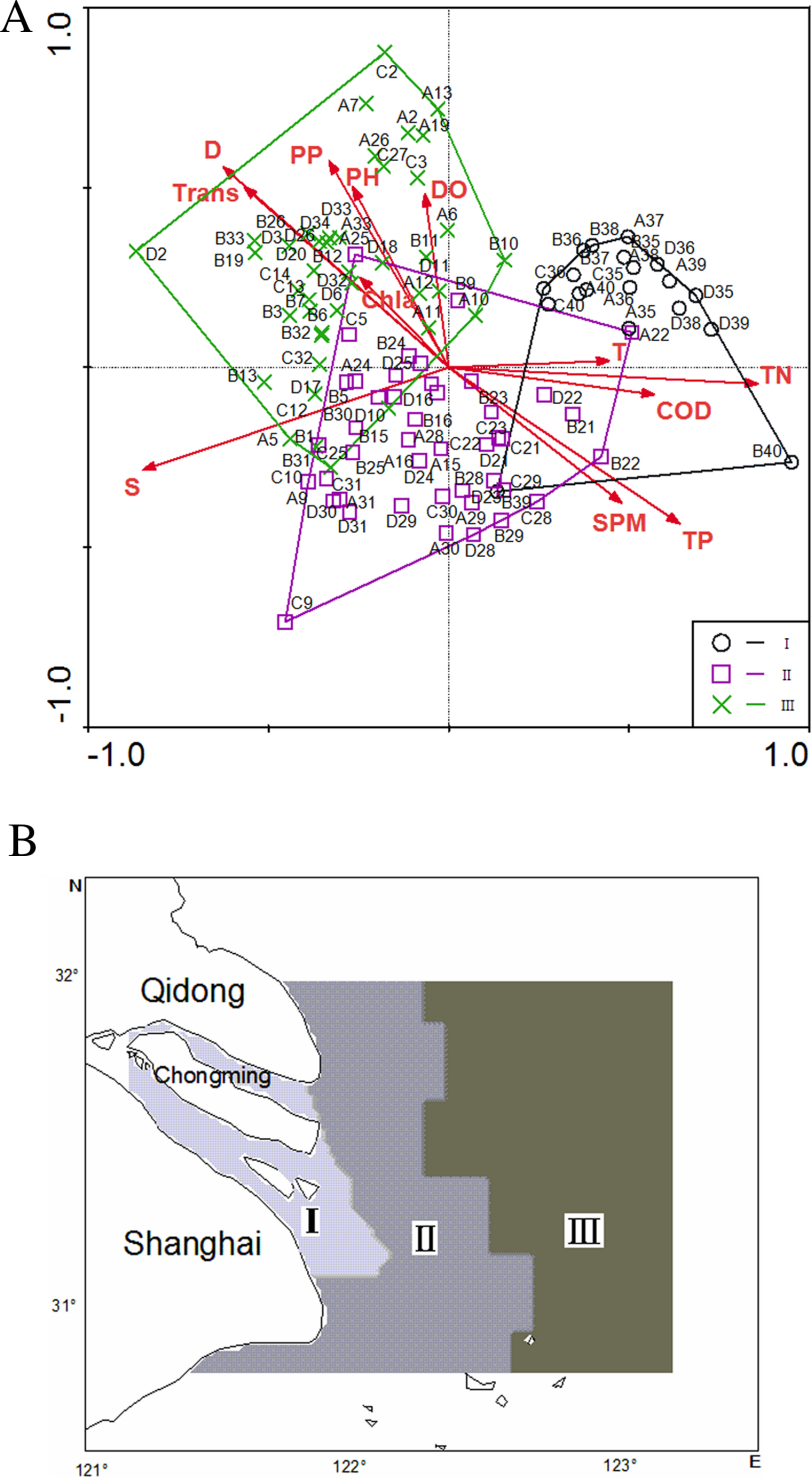
Plot of scores on the first two axes from CCA for sampling stations in the Yangtze Estuary (A, spring in 1999; B, spring in 2001; C, spring in 2004; D, spring in 2007; the numbers following A–D indicate the station numbers)

According to the Monte Carlo tests of *F*-ratios, S (*P* = 0.002), D (*P* = 0.002) and SPM (*P* = 0.008) were the most active environmental parameters affecting ichthyoplankton (Table 6). In addition to these three variables, the other nine environmental variables were evaluated by their inter-set correlations. Any variable having an inter-set correlation coefficient ≥|0.4|, which is the correlation coefficient between site scores (derived from the species scores) and environmental variables, was biologically important. Based on this rule, other environmental variables were inferred as important correlates of one or both of the first two CCA axes. Contrarily, some active environmental variables were weakly correlated with the first two CCA axes. D, S, COD, TP, TN and Trans were strongly correlated with the first axis, and another suite of environmental variables correlated best with the second axis include D and PP (Table 6 and Fig. 3).

**Table 6:** Conditional effect of environmental variables, Canonical coefficients and intra-set correlation of environmental variables with the first two axis of CCA.

Variable	Lambda A	*p*	Coefficient		Correlation	
			Axis 1	Axis 2	Axis 1	Axis 2
D	0.31	**0.002**	0.20	0.68	−**0.51**	**0.43**
S	0.41	**0.002**	−0.52	−0.91	−**0.69**	−0.22
T	0.06	0.490	0.15	−0.01	0.36	0.01
DO	0.15	0.052	0.03	−0.09	−0.05	0.37
pH	0.10	0.150	−0.04	0.22	−0.22	0.39
COD	0.10	0.051	−0.06	0.01	**0.46**	−0.06
TN	0.10	0.096	0.36	−0.06	**0.69**	−0.03
TP	0.15	0.080	0.20	−0.11	**0.52**	−0.33
SPM	0.28	**0.008**	0.21	−0.17	0.39	−0.29
Chla	0.13	0.116	−0.12	−0.27	−0.20	0.19
Trans	0.05	0.644	−0.17	0.04	−**0.46**	0.39
PP	0.08	0.264	0.06	0.39	−0.27	**0.44**

According to the two first axes of the CCA ordination diagram (Fig. 3A) and the sedimentology and eco-hydrological characteristic of different areas (Luo & Shen, 1994), sampling stations can be separated into three discrete groupings: the inner group (I), central group (II) and outer group (III) as shown in Fig. 3B. Different ecological areas stand for different ichthyoplankton assemblages, which differed significantly in their species composition (ANOSIM, Table 7). Each area was characterized by different species (Table 1), as well as different environmental conditions (Table 8).

**Table 7:** *R*-statistic values of species composition of the areas for different years by ANOSIM.

Area	1999	2001	2004	2007
Inner vs. central	0.422^**^	0.741^***^	0.471^**^	0.354^**^
Inner vs. outer	0.451^**^	0.523^***^	0.016	0.155
Central vs. outer	0.405^***^	0.188^**^	0.231^*^	0.075

**Notes.**

* *P* < 0.05.

** *P* < 0.01.

*** *P* < 0.001.

**Table 8:** Environmental data (means and range) of the areas resultant of CCA.

Variable	Inner	Central	Outer
	Mean (range)	Mean (range)	Mean (range)
D (m)	8.37 (5.00–10.00)^A^	12.20 (5.00–32.00)^B^	34.17 (12.00–60.00)^C^
S (‰)	4.03 (2.86–18.03)^A^	19.88 (3.30–28.61)^B^	24.50 (6.62–31.58)^C^
T (°C)	20.10 (18.68–22.22)^A^	18.91 (14.98–21.75)^B^	18.84 (15.89–21.64)^B^
DO (mg/L)	7.64 (5.24–12.03)	6.88 (4.55–10.23)	7.77 (3.90–13.95)
pH	8.13 (7.90–8.36)^A^	8.19 (7.82–8.47)^A^	8.35 (8.06–8.85)^B^
COD (mg/L)	2.80 (1.04–3.52)^A^	2.09 (0.77–4.32)^B^	1.50 (0.46–4.32)^C^
TN (µmol/L)	118.08 (45.90–176.18)^A^	76.62 (34.80–146.87)^B^	49.85 (13.70–86.78)^C^
TP (µmol/L)	2.83 (1.20–7.00)^A^	1.88 (0.55–6.50)^B^	0.74 (0.17–1.50)^C^
SPM (mg/L)	91.75 (28.10–398.00)^A^	163.44 (2.50–1,685.00)^A,B^	5.11 (1.20–30.40)^C^
Chla (mg/m^3^)	0.71 (0.10–2.20)^A^	1.64 (0.10–11.70)^A,B^	2.32 (0.20–17.6)^B^
Trans	0.34 (0.20–0.60)^A^	0.95 (0.20–4.50)^B^	3.37 (0.40–8.50)^C^
PP (mgC/(m^2^∗d))	35.27 (6.70–102.13)^A^	265.04 (7.69–3.213.64)^B^	1,027.07 (64.39–8,326.82)^C^

**Notes.**

Values with different letters (^A–C^) indicate significant difference among years, and values with the same letter indicate that the difference was not significant.

The inner group included the stations in the Estuary, with the shallowest D, the lowest S, the highest T, and the lowest Chla and PP (Table 8). The dominant species was *C. mystus*, which is an anadromous species that migrates to its riverine spawning areas in spring. There were some freshwater species in this group (*Pseudolaubuca sinensis*, *Pseudolaubuca engraulis*) and some adventitious visitors such as *A. bleekeri* (MED), *T. fasciatus* (CA), *Anchoviella commersoni* (ER), and *Setipinna taty* (MED) (Table 9).

**Table 9:** The relative abundance percentage (mean and range) of each species in CCA station group.

Inner		Central		Outer	
*C. mystus*	95.34 (88.99–100)	*A. bleekeri*	44.02 (18.86–59.73)	*E. japonicus*	63.10 (39.58–73.37)
*T. fasciatus*	1.88 (0–7.54)	*T. fasciatus*	21.91 (3.27–44.44)	*C. mystus*	11.56 (0–25.00)
*E. japonicus*	1.53 (0–6.12)	*E. japonicus*	13.73 (1.41–6.11)	*A. bleekeri*	7.14 (0.73–10.42)
*A. bleekeri*	0.87 (0–3.48)	*B. pterotum*	6.12 (0.10–24.38)	*C. hexanema*	4.38 (0–15.45)
*P. sinensis*	0.26 (0–1.05)	*C. mystus*	2.74 (0.97–4.95)	*L. carinatus*	2.60 (0–10.41)
*C. lucidus*	0.09 (0–0.38)	*Gobiidae sp.*	2.56 (0.22–10.00)	*A. commersoni*	2.33 (0–5.00)
*Gobiidae sp.*	<0.01 (0–0.02)	*S. hasta*	2.29 (0–9.16)	*P. polyactis*	1.82 (0–6.25)
*A. commersoni*	<0.01 (0–0.02)	*A. commersoni*	1.25 (0–3.86)	*H. prognathus*	1.27 (0–2.59)
*S. taty*	<0.01 (0–0.02)	*S. marmoratus*	0.99 (0–3.96)	*C. joyneri*	0.93 (0–3.72)
*n.id.2*	<0.01 (0–0.02)	*P. polyactis*	0.84 (0–3.37)	*n.id.1*	0.62 (0–2.50)
		*Sparidae sp.*	0.71 (0–2.83)	*T. japonicus*	0.52 (0–2.08)
		*P. argenteus*	0.60 (0–1.77)	*H. sajori*	0.52 (0–2.08)
		*T. fugu sp.*	0.33 (0–0.71)	*B. pterotum*	0.52 (0–2.08)
		*C. hexanema*	0.24 (0–0.59)	*A. zollingeri*	0.46 (0–1.86)
		*C. stigmatias*	0.22 (0–0.89)	*n.id.3*	0.46 (0–1.86)
		*L. carinatus*	0.14 (0–0.54)	*C. abbreviatus*	0.31 (0–1.24)
		*P. japonicus*	0.13 (0–0.15)	*P. japonicus*	0.25 (0–0.93)
		*P. indicus*	0.12 (0–0.49)	*P. argenteus*	0.23 (0–0.93)
		*F. petimba*	0.12 (0–0.49)	*Anguillidae sp.*	0.23 (0–0.93)
		*E. nebulosus*	0.12 (0–0.48)	*P. indicus*	0.17 (0–0.62)
		*H. prognathus*	0.12 (0–0.48)	*C. stigmatias*	0.15 (0–0.62)
		*C. lucidus*	0.12 (0–0.48)	*J. belengeri*	0.15 (0–0.62)
		*P. engraulis*	0.12 (0–0.48)	*Cynoglossus spp.*	0.07 (0–0.31)
		*L. crocea*	0.12 (0–0.48)	*S. hasta*	0.07 (0–0.31)
		*S. ariskensis*	0.11 (0–0.45)	*S. marmoratus*	0.07 (0–0.31)
		*Sciaenidae sp.*	0.07 (0–0.30)		
		*T. kammalensis*	0.07 (0–0.23)		
		*A. lineatus*	0.06 (0–0.23)		
		*Cynoglossus spp.*	0.01 (0–0.05)		
		*O. rubicundus*	0.01 (0–0.05)		

The central group corresponded to the sampling station with intermediate D and S, and defined the zone of the Estuary with high content of SPM (Table 8). This area consistently contained the highest densities of *A. bleekeri* (MED) and *T. fasciatus* (CA). Other marine-estuarine-dependents (such as *Johnius belengeri*, *Pseudosciaena polyactis*, *Collichthys lucidus*, *Pampus argenteus*), estuarine residents (i.e., *C. hexanema*, *Chaeturichthys stigmatias*, *Gobiidae sp.*, *A. commersoni*, *Thrissa kammalensis*) and some marine species (*E. japonicus*, *Benthosema pterotum*) also used this area as a nursery ground (Table 9).

The outer group covering the deepest sampling stations, with highest S and lowest content of TN and TP, was characterized by the highest Chla and PP (Table 8). It showed great overlap of ichthyoplankton composition with the central group. However, *E. japonicus* (MS) was the key species in the outer area. Other species included *C. mystus* (AN), *A*. *commersoni* (MED), *C. hexanema* (ER), *A. bleekeri*, *Pneumatophorus japonicus* (MS), and *Platycepalus indicus* (MS) (Table 9).

## Discussion

### Ichthyoplankton composition

There are some previous works on ichthyoplankton composition analysis in the Yangtze Estuary. For example, in 1985–1986, ninety-four ichthyoplankton species belonging to 53 families were collected (Yang, Wu & Sun, 1990); in 2000–2003, forty-five ichthyoplankton species belonging to 30 families were collected (Jiang, Shen & Wang, 2006); in 2005–2006, thirty-six ichthyoplankton species were collected (Zhang, Yang & Meng, 2012). In the present study, forty-two ichthyoplankton species belonging to 23 families were collected in four springs, which showed that the ichthyoplankton composition had changed in the Yangtze Estuary over the last 20 years.

The environmental character and nutrient condition of estuarine ecosystems determine the quantity and abundance of ichthyoplankton within them. Most studies have supported the hypothesis that the ichthyoplankton assemblage in estuaries is composed of a few species with high abundance and a large number of rare species, which is a common feature of estuarine populations (Gaughan et al., 1990; Harris & Cyrus, 1995; Whitfield, 1999). The data presented here reinforce this hypothesis. Throughout the study, the four dominant species, *C. mystus*, *E. japonicus*, *A. bleekeri* and *T. fasciatus*, accounted for 88.70% of all the ichthyoplankton.

Furthermore, temperate estuarine ichthyoplankton assemblages have been shown to be dominated by resident species belonging to the Gobiidae family or seasonally by estuarine spawners, such as species in Clupeidae and Engraulidae (Monteleone, 1992; Harris & Cyrus, 2000; Strydom, Whitfield & Wooldridge, 2003; Ramos et al., 2006). Our study of the Yangtze Estuary corroborates these findings. With six species comprising 67.91% of the total catch, we found Engraulidae was the most representative family, followed by Gobiidae with five species and 6.69% of the total catch, Sciaenidae with five species and 0.51% of the total catch, and Cynoglossidae with three species and 0.20% of the total catch. In the Guadiana Estuary (SE Portugal/SW Spain), located in the northern hemisphere at similar latitude as the Yangtze Estuary, Faria, Morais & Chícharo (2006) found that Gobiidae and Engraulidae were the dominant families during spring and summer. Further, their findings on species composition of ichthyoplankton are almost consistent with those from the present study (Faria, Morais & Chícharo, 2006). Although Atherinidae and Cottidae both had only one species in their families, *A. bleekeri* (Atherinidae, 8.61% of the total catch) and *T. fasciatus* (Cottidae, with 13.64% of the total catch) were still the dominant species, so both families were also the most representative. We thus support the notion that Atherinidae is common in estuarine assemblages (Potter et al., 1990). *T. fasciatus* was also a dominant species in Chikugo Estuary in Japan (Islam, Hibino & Tanaka, 2006).

### Inter-annual variability

Estuaries are highly dynamic and their physical and chemical makeup can change over a scale of hours to years (Flint, 1985). Consequently, estuarine fish assemblages, including ichthyoplankton, often exhibit large year-to-year variation in abundance and species. In the present study, environmental condition (for example S, T, DO, pH, TN and TP) had shifted significantly from year to year, but the multivariate similarity analyses on species composition indicated that inter-annual variability of spring ichthyoplankton assemblage structure in the Yangtze Estuary was not pronounced. This suggests that the abovementioned variability of environmental factors had little impact on inter-annual variation of ichthyoplankton assemblage structure in the Yangtze Estuary. With respect to the factors influencing assemblage variations, James, Whitfield & Cowley (2008) established that inter-annual variation may be observed owing to random climatic events such as severe storms, droughts, and cold winters. Longer-term climatic trends, such as El Nino events and climate change, can result in a restructuring of fish assemblages. Selleslagh & Amara (2008) emphasized that the timing of spawning seasons and hence the influx and efflux of individual to and from population, food availability, predation pressure, short-term physicochemical factors (e.g., wind speed and direction, turbidity, wave height, salinity, state of the tide, time of day and temperature) were the factors causing inter-season variations.

However, total CPUE and CPUE for two dominant species (*E. japonicus* and *C. hexanema*) changed markedly in the present study, reflecting the fluctuations in a wide variety of biological and physical variables. The spring of 2001 had the highest ichthyoplankton abundance, which was followed by a significant decrease reaching the lowest abundance in the spring of 2004; a pattern that might be caused by the expansion of the jellyfish *Sanderia malayensis*. Jellyfish are considered to be important zooplanktonic community regulators in several ecosystems (Schneider & Behrends, 1994). According to Xian, Kang & Liu (2005), jellyfish bloomed in the Yangtze Estuary in May 2004, made up 98.44% of total catches, and even covered the surface of the Estuary in some places.

The quality of freshwater inflow is considered a critical ecological factor affecting faunal community structure and species abundance in estuaries (Sklar & Browder, 1998). Changes in river discharge into estuaries and coastal areas can affect nutrient concentrations and ratios, with consequences for primary productivity and associated food webs (Wolanski et al., 2004). In the Yangtze Estuary, the construction of the Three Gorges Dam, South-to-North Water Diversion and Yangtze-Taihu Water Diversion meant that freshwater discharge decreased by 280–1,280 m^3^ s^−1^ (Yu, Jiang & Han, 2007) and the Chla and PP decreased significantly from 2004 to 2007, which showed that the productivity also reduced. Therefore, it is reasonable to hypothesize that low discharge and the reduction of the productivity may have been responsible for reduced ichthyoplankton abundance from 2004 to 2007 by changing the ecological conditions of food and habitat. Similar results have been observed by other scientists. Nixon (2004) proposed that nutrient flow has been restored by human activities he Nile River, including the use of fertilizers for agriculture and the development of sewage systems used by a burgeoning population. Chícharo, Chícharo & Morais (2006) believed changes in salinity and seston, which were mainly due to changes in freshwater input, had an important influence on the structure of the fish assemblages in the Guadiana Estuary (South Portugal).

### Ichthyoplankton assemblage structure and its influencing factors

Ichthyoplankton were distributed in the Yangtze Estuary in three clearly distinct assemblages: the inner area with freshwater covering; the central group, typically brackish; and the outer group with marine influence. Worldwide, this is a common ichthyoplankton distribution pattern in estuaries, such as in the Swan Estuary, the Caete Estuary, the Rio de la Plata Estuary, and in the Guadiana Estuary (Neira, Potter & Bradley, 1992; Faria, Morais & Chícharo, 2006). Several estuarine studies have noted that assemblage structure is shaped by both abiotic and biotic environmental factors (i.e., Whitfield, 1999; Zhu, Liu & Sha, 2002). The suite of environmental variables measured in our study was significantly associated with ichthyoplankton assemblage. As revealed by CCA, the first axis (influenced significantly by D, S, COD, TN, TP and Trans) represented a spatial gradient separating from inner to outer within this estuarine system. Increases in depth, salinity and Trans, and declines in COD, TN and TP were accompanied by changes of ichthyoplankton assemblage structure from the inner, central to outer area.

Salinity is an important determinant of spatial and temporal assemblage structure (Whitfield, 1999). Salinity varies from low to high owing to the Estuary’s physiography and river flow inputting, so areas with different salinity support ichthyoplankton assemblages belonging to different ecological guilds. Islam & Tanaka (2006) identified four different spatial distribution patterns of larval and juvenile fishes: oligohaline, mesohaline, euryhaline and polyhaline. Here we demonstrate that the inner area was dominated by anadromous (*C. mystus*) and freshwater (*P. sinensis* and *P. engraulis*) species, the central area featured brackish species (*A. bleekeri* and *T. fasciatus*), while the outer area was dominated by marine species, such as *E. japonicus*, *B. pterotum*, and *P. argenteus*.

Depth varying from inner to outer areas is one of the most important environmental characters in estuarine ecological systems. Our results show that depth has a stronger influence on the spatial structure of the Yangtze Estuary’s ichthyoplankton assemblage. This is likely related to the depth preferences of different taxonomic groups, as found in other regions (Sassa et al., 2004; Muhling, Beckley & Olivar, 2007). Larvae from families such as the Engraulidae and Myctophidae were found at shallower depths than those from the Gobiidae and Cynoglossidae. Furthermore, it is well documented that the depth distribution of a species can differ depending on the size and age of the larvae (Loeb, 1979).

Measures of suspended matter particulate can be used to determine the estuarine turbidity maximum (ETM) location (Herman & Heip, 1999). A special high SPM content zone stood out in the central Estuary of the Yangtze Estuary and adjacent coastal water, where the ETM exists all year round. ETM has been identified as an important nursery area for the ichthyoplankton assemblage comprising *A. bleeker, T. fasciatus*, *B. pterotum*, and *T. fugu sp.* Several hypothesis may explain this distribution. (a) The higher prey concentration in the ETM is advantageous for feeding in larval and juvenile fishes (Islam, Hibino & Tanaka, 2006). (b) Larval and juvenile fish predators encounter more prey under conditions of elevated turbidity owing to turbulence-induced encounter rates (Rothschild & Osborn, 1988). (c) The ETM offers increased survival rates because predators of larval fishes are generally less frequented in these regions (Parrish, 1989).

Besides D, S, and SPM, several other parameters such as COD, TN, TP, Trans and PP in this study also affect spatial organization of the ichthyoplankton assemblage. TN, TP and PP represented nutrient condition, while COD and Trans indicated water quality. Jiang, Shen & Wang (2006) and Jiang, Shen & Chen (2006) suggested that an abundance of nutrients brought by the Yangtze River flow played an active role in the spawning of adult fishes and hatching of eggs, but also influenced the distribution of larval and juvenile marine fishes. Yang, Wu & Sun (1990) proposed that unpolluted water was the principal and basic condition favoring reproduction and growth of fishes.

Variation in species distribution explained by the first four axes of CCA was only 15.3%, which indicates that other factors could influence the ichthyoplankton assemblage in the Yangtze Estuary. Another potential factor driving species distribution in estuarine assemblages may be reproductive biology. The effect of these variables on ichthyoplankton assemblage structure needs to be determined by further investigations in the Yangtze Estuary.

## Conclusions

The ichthyoplankton assemblage in the Yangtze Estuary is composed of a few species with high abundance and a large number of rare species, which is a common feature of estuarine populations. Across all four springs, the four dominant species were *C. mystus*, *E. japonicus*, *A. bleekeri* and *T. fasciatus*. Temperate estuarine ichthyoplankton assemblages have been shown to be dominated by resident species belonging to Gobiidae family or seasonally by estuarine spawners, such as species in Clupeidae and Engraulidae, and the Yangtze Estuary shows a similar pattern.

In the present study, environmental conditions such as S, T, DO, pH, TN and TP shifted significantly from year to year, but multivariate similarity analyses on species composition indicated that the inter-annual variability of spring ichthyoplankton assemblage structure in the Yangtze Estuary was not pronounced. This suggests that the abovementioned environmental variability had little impact on inter-annual variation of ichthyoplankton assemblage structure in the Yangtze Estuary.

The suite of environmental variables measured in our study was significantly associated with ichthyoplankton assemblage. As revealed by CCA, the first axis (influenced significantly by D, S, COD, TN, TP and Trans) represented a spatial gradient separating from the inner to outer area within this estuarine system. Increases in depth, salinity and Trans, and declines in COD, TN and TP were accompanied by a change of ichthyoplankton assemblage structure from the inner, central to outer area.

## Supplemental Information

10.7717/peerj.1186/supp-1 Supplemental Information 1 Raw data of environment

10.7717/peerj.1186/supp-2 Supplemental Information 2 Raw data of species
